# Utility of prenatal ultrasound in diagnosis and prognosis of aortic arch obstruction: a 10-year single-center retrospective study

**DOI:** 10.3389/fmed.2026.1736882

**Published:** 2026-07-03

**Authors:** He Li, Jinwen Chen, Wen Ling, Qiumei Wu, Biying Huang, Guorong Lyu, Caihong Jiang, Zongjie Weng

**Affiliations:** 1 Department of Medical Ultrasonics, Fujian Maternity and Child Health Hospital, College of Clinical Medicine for Obstetrics and Gynecology and Pediatrics, Fujian Medical University, Fuzhou, China; 2 Department of Medical Ultrasonics, The Second Affiliated Hospital of Fujian Medical University, Quanzhou, China

**Keywords:** aortic arch obstruction, echocardiography, fetus, postnatal analysis, prenatal diagnosis

## Abstract

**Objective:**

This study aimed to evaluate the prenatal echocardiographic features, diagnostic accuracy, associated anomalies, and early postnatal outcomes of fetal aortic arch obstruction, with particular emphasis on differentiating interrupted aortic arch from coarctation of the aorta.

**Methods:**

We conducted a retrospective analysis of 72 cases of aortic arch obstruction over a 10-year period, from 1 June 2014 to 1 June 2024, including interrupted aortic arch (IAA) and coarctation of the aorta (CoA). Prenatal ultrasound and medical records were used to summarize the characteristic ultrasound manifestations, genetic characteristics, and combined malformations, and to track perinatal and clinical outcomes, including postpartum echocardiography or CTA and other imaging examinations, surgical procedures, and prognosis.

**Results:**

A total of 72 fetal cases were retrospectively analyzed, including IAA types A, B, and C (22.22, 20.83, and 6.94%, respectively) and CoA (50.00%). The prenatal diagnostic accuracy was 90.28%. The IAA group had significantly smaller aortic valve annulus Z-scores, ascending aorta Z-scores, and aortic-to-pulmonary artery diameter ratios but significantly larger left-to-right ventricular (LV-to-RV) diameter ratios, ventricular septal defect-to-aortic diameter ratios, and ductus arteriosus Z-score compared to CoA (*p* < 0.05). Intracardiac malformations occurred in 88.89% of cases, with the prevalence of combined anomalies significantly higher in IAA (*p* < 0.001). Clinically, IAA cases showed more severe hypoxia and cyanosis and lower postoperative survival, whereas those with CoA cases had favorable outcomes.

**Conclusion:**

Prenatal echocardiography is valuable not only for detecting fetal aortic arch obstruction but also for differentiating IAA from CoA and guiding perinatal risk stratification. IAA is associated with more complex intracardiac anomalies, greater ductal dependence, and poorer early outcomes, whereas CoA shows greater clinical heterogeneity and requires close postnatal surveillance.

## 1 Introduction

Aortic arch obstruction, such as coarctation of the aorta (CoA) and interrupted aortic arch (IAA), is a prevalent congenital heart defect (1). CoA accounts for approximately 7% of all live-born cases of congenital heart diseases (2), whereas IAA is less common, with an incidence of 19 per million live births, and accounts for 1% of all congenital heart defects (3). CoA is defined as the congenital narrowing of the descending aorta, which results in a smaller lumen. Based on the location of the narrowing relative to the ductus arteriosus, CoA can be classified into preductal and postductal types. Prenatal ultrasound is primarily used to diagnose the preductal type. IAA is characterized by the complete interruption of the continuity between the ascending aorta and the descending aorta. According to the classification criteria established by Celoric and Patton, IAA can be categorized into three distinct types: Type A, B, and C (43, 53, and 4%, respectively) (4). Aortic arch obstruction is seldom encountered in isolation and is frequently associated with other intracardiac abnormalities, particularly ventricular and atrial septal defects, complete transposition of the great arteries, and double-outlet right ventricles (5–7). Furthermore, aortic arch obstruction, particularly type B interrupted aortic arch, is strongly associated with chromosomal abnormalities, particularly 22q11.2 deletion syndrome, which may manifest with the features of DiGeorge syndrome (8, 9). Postnatally, neonates with isolated CoA often retain varying degrees of antegrade flow or develop collateral circulation and thus typically do not present with severe hypoxia immediately at birth. In contrast, IAA represents a complete anatomical discontinuity, resulting in a profoundly duct-dependent systemic circulation that is absolutely incompatible with survival without continuous prostaglandin E1 (PGE1) infusion and urgent surgical intervention. Consequently, accurate prenatal differentiation between IAA and CoA is paramount for early risk stratification and neonatal prognosis.

While postnatal surgical interventions—such as autologous vascular patch repair and end-to-end anastomosis—have significantly improved survival rates in infants with aortic arch obstruction (10–14), favorable clinical outcomes depend on an integrated continuum of care that includes accurate prenatal diagnosis, appropriate delivery planning, prompt postnatal recognition and stabilization, and timely neonatal cardiac management. To enhance diagnostic precision, recent studies have explored various advanced echocardiographic modalities, such as three- and four-dimensional (3D/4D) ultrasound (15–18), high-definition Doppler for capturing aberrant blood flow (19, 20), fetal cardiac strain analysis for quantifying ventricular dysfunction (2, 21), and artificial intelligence-assisted automated image analysis (22). Despite these technological strides, the routine prenatal detection rate of aortic arch obstruction remains suboptimal (21.7–52%), with the false-positive rate for CoA reaching approximately 50% (23, 24). A major contributing factor to this diagnostic bottleneck is that the routine sonographic manifestations of IAA and CoA can be strikingly similar, which make their accurate prenatal differentiation a persisting clinical challenge (25).

Although both IAA and CoA belong to the spectrum of fetal aortic arch obstruction, they differ substantially in anatomical severity, the degree of ductal dependence, requirements for immediate postnatal management, and prognosis. In particular, IAA generally requires prompt neonatal cardiovascular assessment and stabilization, maintenance of ductal patency with prostaglandin E1, and early surgical repair, whereas the timing and intensity of intervention for CoA vary according to the severity of the obstruction, ductal status, and the infant’s clinical condition. Prenatal differentiation between these two entities remains challenging because both may present with a small aortic arch, ventricular disproportion, and an abnormal three-vessel-trachea view. Therefore, a systematic comparison of prenatal echocardiographic findings, associated anomalies, genetic results, and early postnatal outcomes may help improve prenatal counseling and perinatal management. In this study, we retrospectively analyzed fetuses diagnosed with aortic arch obstruction over a 10-year period at a single tertiary center. The aim was to evaluate prenatal echocardiographic features, diagnostic accuracy, associated anomalies, and early outcomes, with emphasis on differentiating IAA from CoA.

## 2 Materials and methods

### 2.1 Study population

A total of 134,689 fetuses underwent systematic prenatal ultrasonography at our center between 1 June 2014 and 1 June 2024. For the present study, cases were screened from the prenatal ultrasound database. To systematically identify cases with discordant prenatal and final diagnoses, such as false-negative and prenatally misclassified cases, postnatal echocardiographic, computed tomography angiographic, and pediatric surgical follow-up records at our center were cross-referenced with the prenatal ultrasound database. This process identified infants in whom aortic arch obstruction had not been suspected prenatally, as well as those in whom a prenatal cardiovascular diagnosis was inconsistent with the final postnatal diagnosis. A false-negative case was defined as a fetus in whom aortic arch obstruction was not suspected prenatally but was subsequently confirmed after birth. A prenatally misclassified case was defined as one in which a cardiovascular abnormality was detected prenatally, but the specific prenatal diagnosis was inconsistent with the final postnatal diagnosis. These cases were collectively classified as cases with discordant prenatal and final diagnoses. For these cases, the timing and indication for postnatal evaluation, clinical presentation, confirmatory imaging, treatment, surgical intervention, survival, and follow-up status were reviewed. Eligible cases had prenatal ultrasound examinations performed at our center with adequate data available for retrospective evaluation of the aortic arch. For terminated fetuses, the final diagnosis was confirmed by pathology, and for live-born infants, the final diagnosis was confirmed by postnatal echocardiography, CTA, and/or surgical findings. Live-born infants were followed up at 3 and 6 months after birth and annually thereafter. IAA was defined as a complete anatomical discontinuity between the ascending and descending aorta. Thirty-six cases with confirmed IAA met the eligibility criteria and were included. CoA was defined as localized or segmental narrowing of the aortic isthmus or juxtaductal aortic arch with preserved continuity between the ascending and descending aorta. During the same study period, 36 consecutive cases with confirmed CoA met the eligibility criteria and were included in the CoA group. Both isolated and non-isolated CoA cases were eligible for inclusion. Associated intracardiac and extracardiac anomalies were not used as the exclusion criteria in order to reflect the clinical heterogeneity of CoA. Exclusion criteria were suspected, but unconfirmed IAA or CoA, incomplete prenatal imaging data, insufficient pathological or postnatal confirmation, and other aortic arch abnormalities could not be definitively classified as IAA or CoA. Prenatally suspected CoA cases that ended in termination of pregnancy were excluded when definitive pathological confirmation was unavailable because mild or evolving isthmic narrowing is difficult to establish reliably in mid-trimester specimens. Therefore, the final CoA cohort consisted of live-born infants with definitive postnatal confirmation and included both prenatally diagnosed and prenatally missed cases, provided that adequate prenatal ultrasound data were available for a retrospective review. In the IAA group, the mean maternal age was 29.03 ± 3.94 (21–43) years, and the mean gestational age was 23.41 ± 2.10 (17.14–29.00) weeks. The mean maternal age of the CoA group was 31.36 ± 4.86 (20–40) years, and the mean gestational age was 25.90 ± 4.28 (20.57–39.00) weeks (Table 1). We followed up on all subjects by telephone at 3 and 6 months postpartum and annually thereafter. Live births confirmed by prenatal ultrasound diagnosis were reconfirmed by postnatal echocardiography, CTA, or cardiac surgery. This study was approved by the Ethics Committee of Fujian Maternity and Child Health Hospital, Fujian Medical University (approval number: 2025KY027), and informed consent was obtained from all patients. In cases of abortion, the family signed an informed consent form for autopsy review.

**Table 1:** Baseline characteristics of fetuses with IAA and CoA.

Characteristic	IAA (*n* = 36)	CoA (*n* = 36)	*p* value
Maternal age (y)	29.03 ± 3.94	31.36 ± 4.86	0.028
Gestational age at diagnosis (wk)	17.14–29.00	20.57–39.00	0.003
Isolated	0	8	0.009
Non-isolated	36	28	
Intracardiac anomalies	36	19	<0.001
Extracardiac anomalies	8	15	0.129
Normal genetic detection^a^	12	11	1.000
Abnormal genetic detection^b^	3	3	
Clinical outcome			
Termination of pregnancies	28	0	<0.001
Live births	8	36	<0.001
Symptomatic	8	6	<0.001
Asymptomatic	0	30	<0.001
Surgery	5	8	0.042
Postoperative complication	2 (40.0%)	1 (12.5%)	0.510
Surgical mortality	1 (20.0%)	1 (12.5%)	1.000

^a^Normal genetic detection indicates that all performed genetic tests (chromosomal karyotyping and/or SNP array analysis) yielded normal results. ^b^Abnormal genetic detection indicates that a pathogenic genetic abnormality was identified by either chromosomal karyotyping or SNP array analysis.

### 2.2 Fetal echocardiography

**Figure 1:**
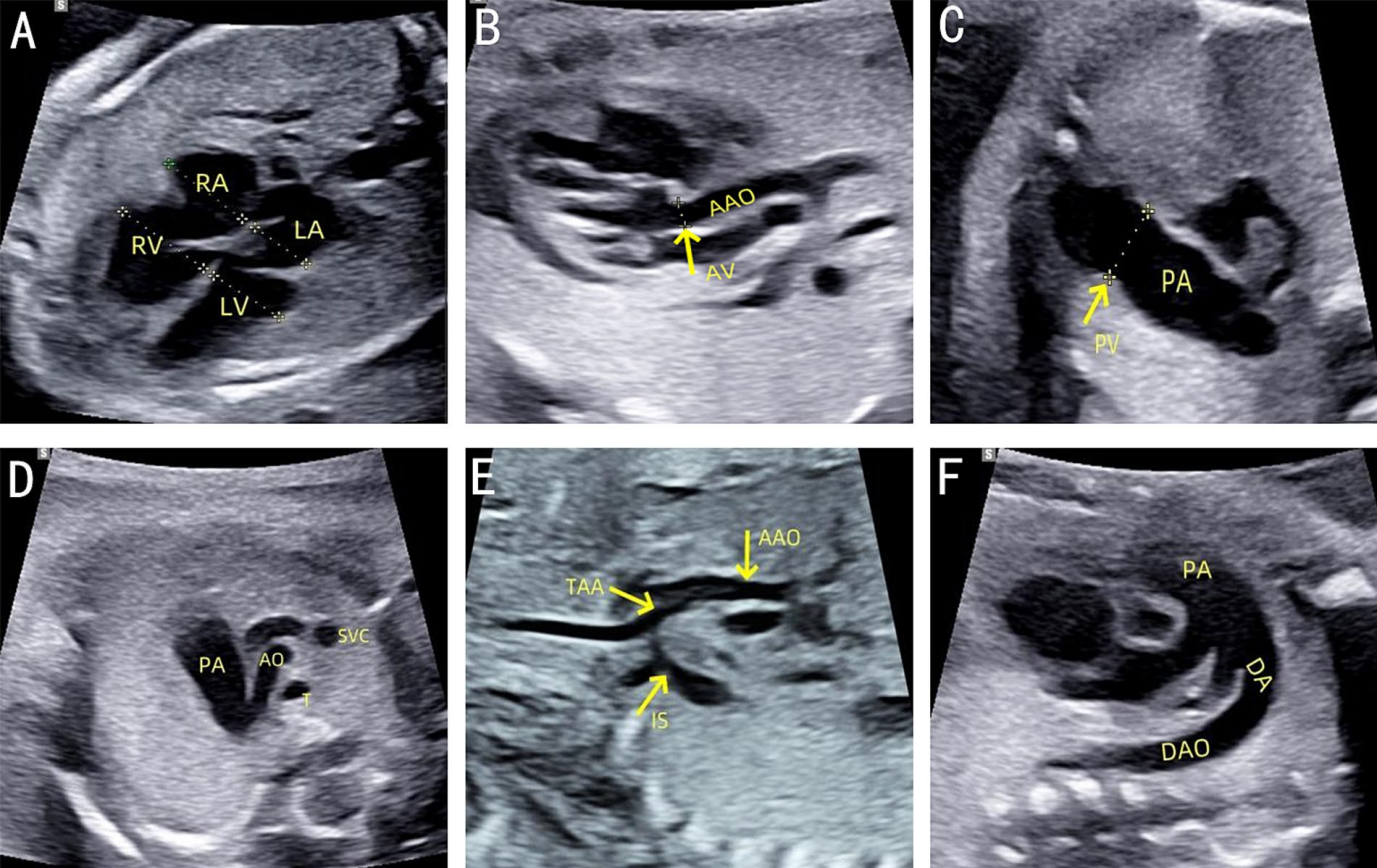
Main observation and measurement sections. **(A)** Four-chamber heart section: Observe the ratio of the left and right hearts, and measure the internal diameters of the left atrium, left ventricle, right atrium, and right ventricle. **(B)** Left ventricular outflow tract section: Observe for ventricular septal defect and measure the inner diameter of the aortic valve annulus. **(C)** Right ventricular outflow tract section: measurement of pulmonary valve annulus inner diameter. **(D)** Three-vessel tracheal section: measurement of the main pulmonary artery and aortic diameter. **(E)** Aortic arch cross-section: Measure the internal diameter of the ascending aorta, transverse aortic arch, isthmus of the aortic arch, and descending aorta. **(F)** Arterial duct arch section: Measure the inner diameter of the arterial duct.

Fetal cardiac screening was conducted in accordance with the stringent guidelines of the International Society of Ultrasound in Gynecology and Obstetrics (ISUOG), utilizing advanced color Doppler ultrasound diagnostic equipment, such as the GE Voluson S8, E8, E10, Samsung W10, and Philips EPIQ 7C (26). The transducer frequency was set between 4.0 and 8.0 MHz. We used a nine-section methodology to comprehensively analyze the fetal heart. In suspected cases of aortic arch obstruction, we focused on the four-chamber, three-vessel, aortic arch, and long-axis views of the ductal arch and measured the sizes of the left and right atria, the left and right ventricles, ventricular septal defect, aortic ring, pulmonary valve ring, main pulmonary artery, ascending aorta (middle), transverse aortic arch, isthmus of the aortic arch, descending aorta, and ductus arteriosus. The Z-scores of the aortic valve annulus inner diameter, ascending aorta (mid-segment), descending aorta, pulmonary valve annulus inner diameter, pulmonary artery trunk, left ventricular transverse diameter, right ventricular transverse diameter, and the ductus arteriosus, as well as the ratios of the left ventricular transverse diameter to the right ventricular transverse diameter and those of the aortic inner diameter to the pulmonary artery inner diameter, were calculated (Figure 1). A careful examination was performed to determine whether there were other intra- or extracardiac abnormalities for fetuses with aortic arch obstruction detected by ultrasound.

### 2.3 Neonatal imaging

Postpartum echocardiography was performed using the Philips EPIQ 7C and IE Elite diagnostic ultrasound machine, with a probe frequency of 3.0–8.0 MHz, in accordance with the American Society of Echocardiography (ASE) Guidelines for Pediatric Echocardiography (27). A comprehensive heart scan was performed using segmental analysis to assess the origin, internal diameter, and blood flow of the aorta and its branches. In suspected cases of aortic arch obstruction, the ultrasound focus was directed toward the parasternal long-axis view, short-axis view of the great arteries, five-chamber view, and suprasternal notch view.

Neonates were sedated and placed in the supine position for examination using the GE Revolution 256-row CT scanner. Scanning was performed from the root of the neck to 4 cm below the diaphragm. Initially, routine plain scanning was performed, followed by CTA. The scanning parameters were set at 80–100 kV and 110–300 mA, and the volumetric scanning technique was used (with the ability to reconstruct images with a layer thickness of 0.625 mm). The thickness layer was maintained at 3 mm. A high-pressure syringe was automatically pushed at a flow rate of 1.0–1.5 mL/s. Iopromide (370 mgI/mL), the contrast agent, was administered at a dose of 1.0–1.5 mL/kg. Scanning delay time was determined using the threshold method, with the ROI placed in the root of the aorta or pulmonary artery trunk, and the threshold value set at 100 HU. Scanning was automatically triggered when the contrast agent reached 100 HU. The raw reconstruction thin-layer CT data were transferred to the Advantage Windows 4.6 workstation for multiplanar reconstruction (MPR), CT Volume Rendering (CTVR), minimum density projection (MIP) reconstruction, and 3D aortic reconstruction (28). In suspected cases where aortic arch obstruction was detected, key areas of observation were located in the ascending aorta and aortic arches.

### 2.4 Genetic examination

All pregnant women who were diagnosed with aortic arch obstruction through ultrasound scanning were provided with comprehensive counseling on the diagnosis and treatment options available. Some women underwent further chromosomal karyotyping and/or single-nucleotide polymorphism (SNP) microarray analysis of fetal amniotic fluid or umbilical cord blood to screen for any underlying genetic abnormalities in the fetus. In this study, “normal genetic detection” was defined as normal results yielded from all performed genetic tests (chromosomal karyotyping and/or SNP array analysis). Conversely, “abnormal genetic detection” was defined as the presence of a pathogenic genetic abnormality identified by either chromosomal karyotyping or SNP array analysis.

### 2.5 Prenatal counseling and perinatal management

After prenatal suspicion or diagnosis of aortic arch obstruction, all cases were reviewed by experienced fetal cardiologists. The assessment included detailed fetal echocardiography, evaluation of intracardiac and extracardiac anomalies, and recommendations for genetic counseling and chromosomal testing when clinically indicated. Families received counseling regarding the fetal cardiac diagnosis, associated anomalies, possible genetic abnormalities, expected postnatal course, and available treatment options. Pregnancy management, including continuation or termination of pregnancy, was determined by the parents following counseling, according to fetal cardiac anatomy, associated anomalies, genetic findings, gestational age, and parental preference. For continuing pregnancies, delivery at a tertiary center with neonatal cardiovascular evaluation and prostaglandin availability was recommended, particularly for suspected ductal-dependent lesions, such as interrupted aortic arch.

### 2.6 Statistical analysis

SPSS software (version 26.0) was used for data analysis. Continuous variables were expressed as mean ± standard deviation (SD). A two-sided independent-samples *t*-test was used to compare normally distributed continuous variables between the two groups. The Mann–Whitney U test was used for data that were not normally distributed, and the data were presented as medians and interquartile ranges [M (IQR)]. The Kruskal–Wallis test was used for comparing multiple groups. Statistical significance was determined using a two-sided test. Statistical significance was set at a *p-*value of < 0.05. Therefore, a *p*-value of < 0.05 indicated a significant intergroup difference.

## 3 Results

### 3.1 Study cohort and final diagnosis

A total of 72 fetuses with confirmed aortic arch obstruction were included in the final analysis. These consisted of 36 cases of IAA and 36 cases of CoA. Among the IAA cases, 16 were type A, 15 were type B, and 5 were type C. Final diagnosis was confirmed by pathological autopsy in 28 terminated cases and by postnatal echocardiography, CTA, and/or surgical findings in 44 live-born infants. Overall, 28 pregnancies were terminated, and 44 infants were born alive. Among these 44 infants, there were four (9.09%) cases of IAA type A, three (6.82%) of IAA type B, and one (2.27%) of IAA type C, as well as 36 cases of coarctation of the aorta (81.82%). Prenatal and final diagnoses were concordant in 65 of the 72 cases (90.28%). Seven cases (9.72%) showed discordance between the prenatal and final diagnoses, comprising five false-negative CoA cases and two prenatally misclassified IAA cases.

### 3.2 Prenatal echocardiographic features of aortic arch obstruction

**Figure 2:**
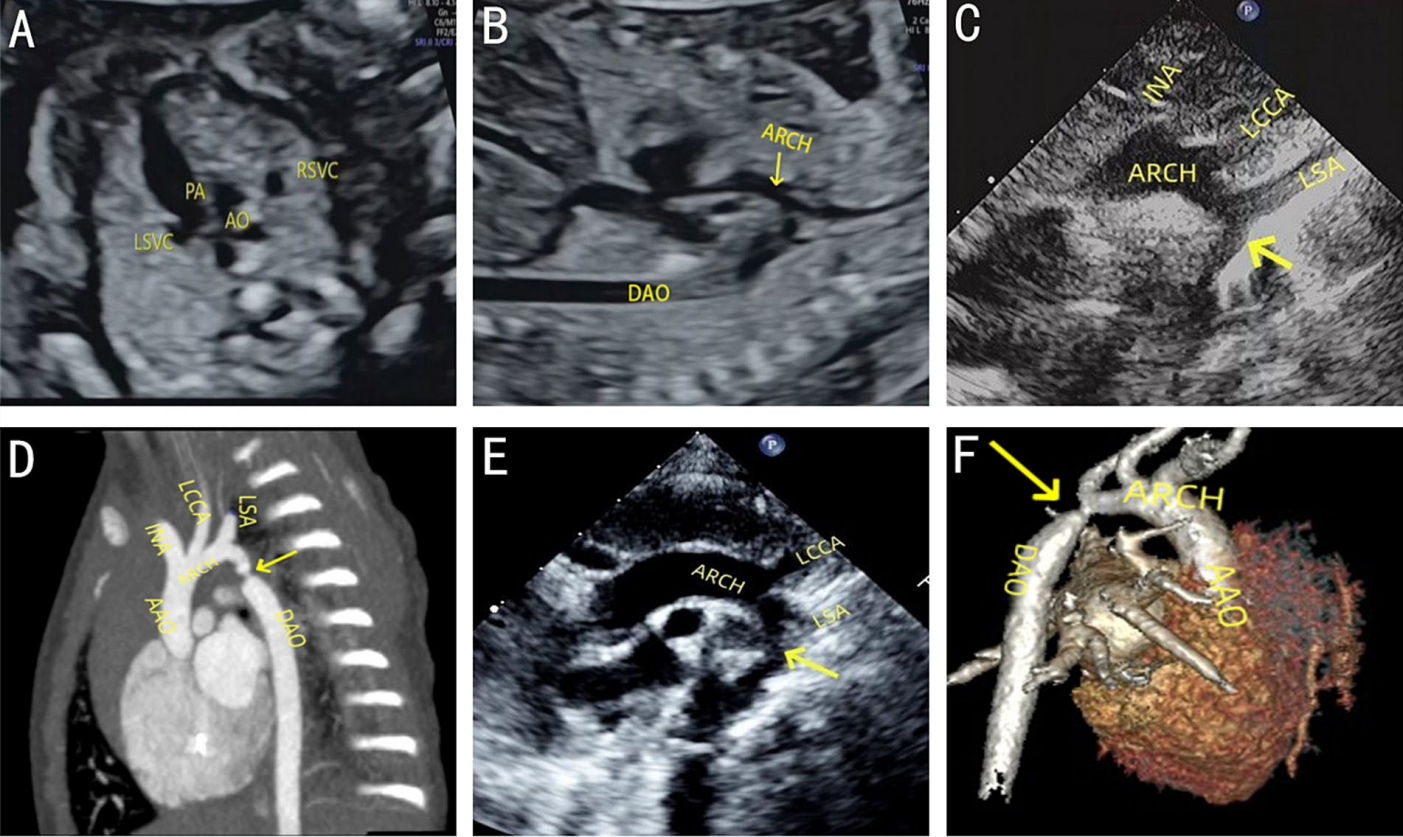
CoA prenatal ultrasound and postnatal imaging. **(A)** The pulmonary artery is larger than the aorta, and the superior vena cava are double. **(B)** The aortic arch is tortuous, rigid, and narrow. **(C)** Preoperative ultrasound showed narrowing of the descending aorta. **(D)** Preoperative CTA showed narrowing of the descending aorta. **(E)** Postoperative ultrasound showed residual obstruction in the descending aorta. **(F)** Postoperative follow-up CT three-dimensional reconstruction of residual obstruction in the descending aorta.

**Figure 3:**
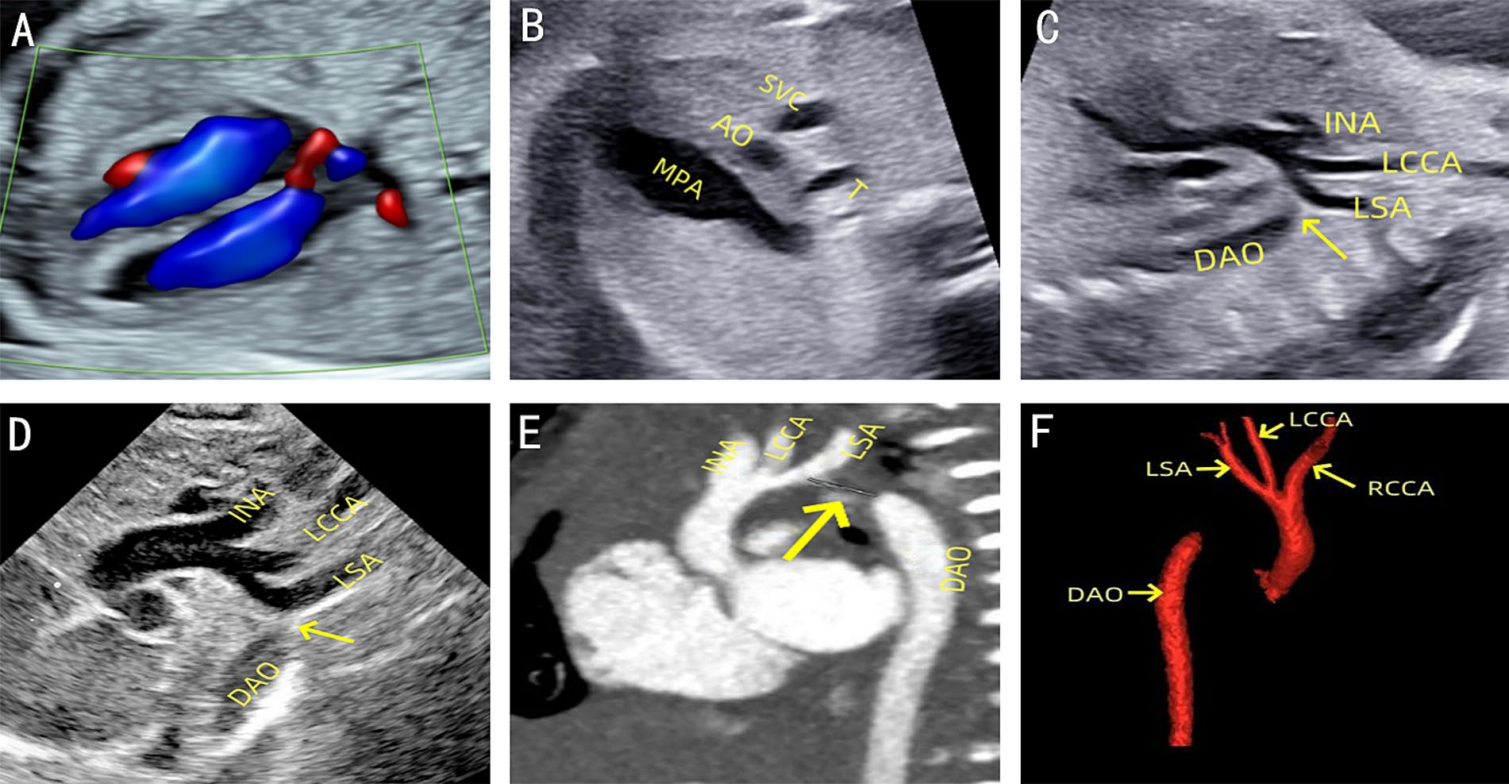
IAA Type A Prenatal Ultrasound and Postnatal Imaging. **(A)** The left and right heart proportions are basically symmetrical. **(B)** The pulmonary artery is significantly larger than the aorta, and the internal diameter of the aorta is slightly smaller than that of the superior vena cava. **(C)** The aortic arch gives off the left subclavian artery and then breaks continuity with the descending aorta. **(D)** Postpartum echocardiogram, arrow indicates interruption location. **(E)** Postpartum CTA imaging, with arrows indicating the location of the interruption. **(F)** Postpartum CTA three-dimensional reconstruction.

**Figure 4:**
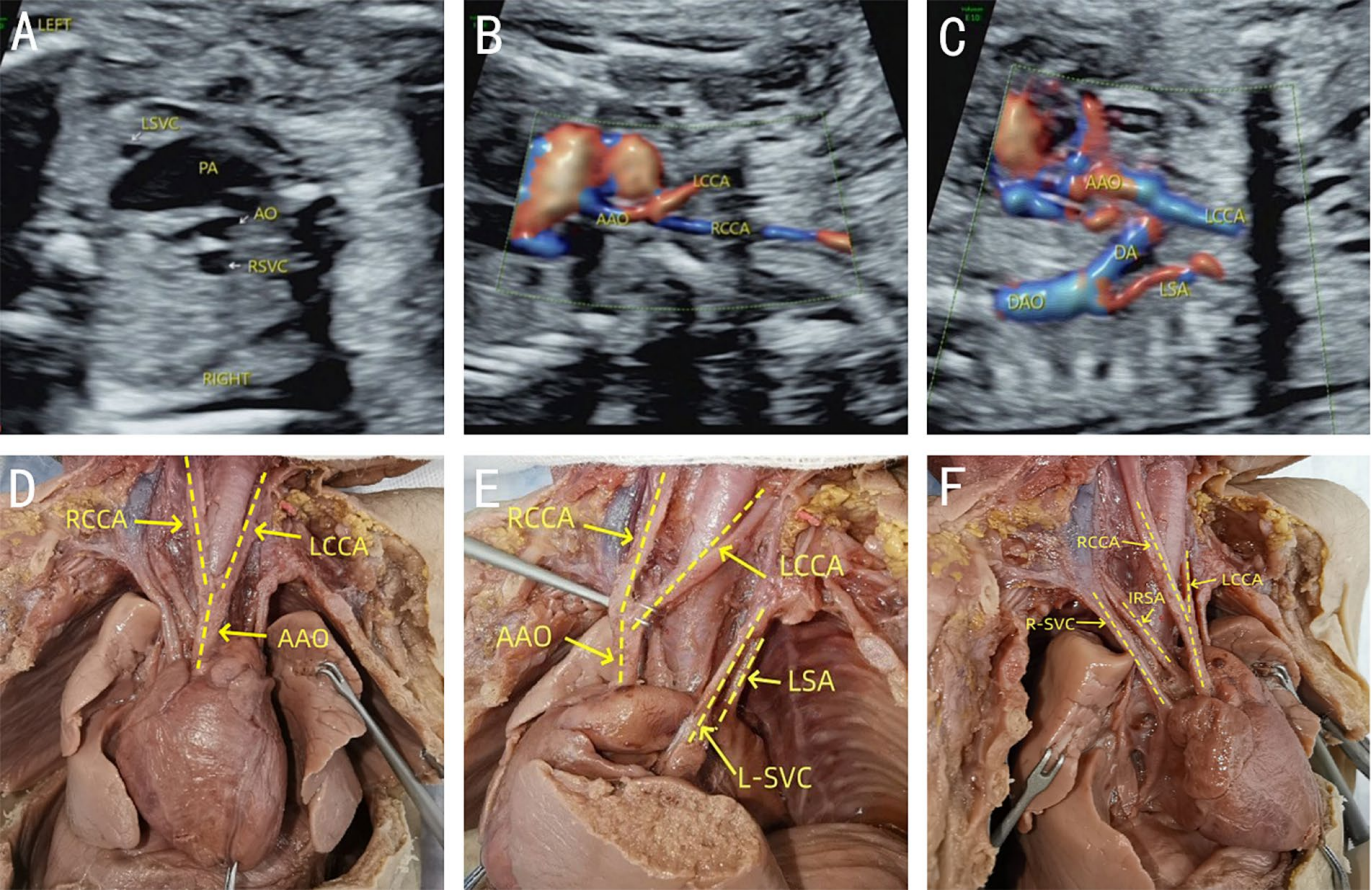
IAA Type B prenatal ultrasound and postpartum local pathological anatomy. **(A)** The pulmonary artery is significantly enlarged, and the diameter of the aorta is smaller than that of the pulmonary artery. **(B)** The ascending aorta is discontinuous with the descending aorta after it has given rise to two branches. **(C)** The left subclavian artery is connected to the descending aorta. **(D–F)** Following induced abortion, local pathological findings revealed malformation of the ascending aorta. After the ascending aorta gives rise to the right common carotid artery and the left common carotid artery, it loses continuity with the descending aorta. The right subclavian artery connects to the pulmonary artery, and double superior vena cavae are visible.

**Figure 5:**
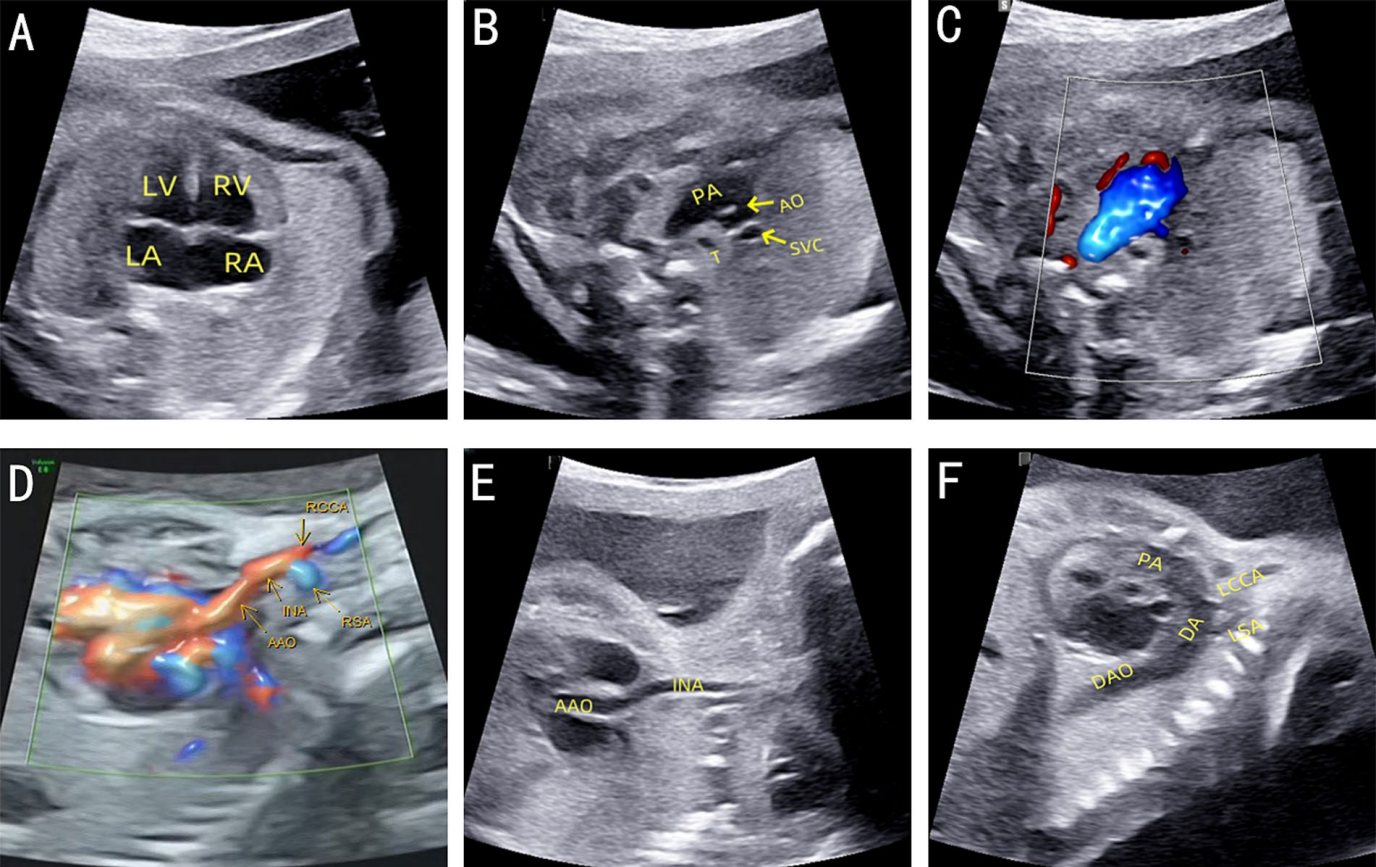
IAA Type C Prenatal Ultrasound. **(A)** The left and right heart proportions are basically symmetrical. **(B,C)** The pulmonary artery is significantly larger than the aorta, and the internal diameter of the aorta is slightly smaller than that of the superior vena cava. **(D,E)** The aortic arch continues only as the innominate artery. **(F)** The left common carotid artery and left subclavian artery connect to the descending aorta via the arterial duct.

In our cohort, common prenatal echocardiographic features of IAA and CoA included a significantly smaller aortic diameter compared to the pulmonary artery diameter on the three-vessel view. The sagittal aortic arch view failed to show the normal ‘candy-cane’ appearance, presenting instead with a rigid course. Differentiation between the two entities relied primarily on anatomical continuity: IAA was characterized by the loss of the normal ‘V’ shape in the three-vessel cross-section, with complete anatomical discontinuity and blood-flow interruption between the ascending and descending aorta. CoA was characterized by localized stenosis of the aortic isthmus (Figure 2). The ultrasonographic characteristics of IAA were based on the different positions of disconnection: Type A (disjunction distal to the left subclavian artery) was characterized by the cephalic extension of the aorta while branching in a ‘W’ shape, with the blind end of the descending aorta and the absence of the left subclavian artery transition segment (Figure 3); type B (disjunction between the left common carotid and the left subclavian artery) was characterized by the cephalic extension of the aorta while branching in a ‘Y’ shape, with the arch terminating at the level of the left common carotid artery branch (Figure 4); and type C (disjunction between the innominate artery and the left common carotid artery) was characterized by the cephalic extension of the aorta while branching in an ‘I’ shape; this rare subtype is frequently associated with an abnormal origin of the right subclavian artery (Figure 5). In the comparison between the IAA and CoA groups, the aortic valve annulus Z-score, ascending aorta Z-score, and aortic-to-pulmonary artery diameter in the IAA group were significantly smaller than those in the CoA group (*p* < 0.05), while the left ventricular-to-right ventricular diameter, ventricular septal defect size-to-aortic diameter, and ductus arteriosus Z-score were significantly larger than those in the CoA group (*p* < 0.05; Table 2). A chi-square test of the various parameters of different IAA subtypes revealed that there were no significant differences in any of the parameters (Table 3).

**Table 2:** Heart dimensions and vessel measurement in IAA and CoA.

Echocardiographic parameter	IAA (*n* = 36)	CoA (*n* = 36)	*p* value
Z-score of LVD	−1.46 (−2.79, −0.75)	−2.02 (−3.04, −1.19)	0.177
Z-score of RVD	−1.03 (−1.92, 0.06)	−0.01 (−1.31, 0.88)	0.054
LV/RV	0.84 (0.70, 0.96)	0.75 (0.61, 0.88)	0.037
VSD/AO	1.50 (1.31, 1.84)	0.00 (0.00, 0.52)	<0.001
Z-score of AVA	−3.24 (−4.41, −2.08)	−1.08 (−2.46, −0.37)	<0.001
Z-score of PVA	1.67 (0.02, 2.34)	0.93 (−0.80, 1.85)	0.205
Z-score of MPA	0.97 (0.43, 1.80)	0.67 (0.21, 1.10)	0.084
Z-score of AA	−3.69 (−4.60, −2.90)	−1.67 (−2.50, −0.97)	<0.001
Z-score of DAO	−0.07 (−1.39, 0.83)	−0.39 (−1.45, 0.64)	0.347
Z-score of DA	0.89 (0.01, 1.69)	0.12 (−1.20, 0.91)	0.018
AO/PA	0.46 (0.41, 0.55)	0.64 (0.57, 0.71)	<0.001

**Table 3:** Heart dimensions and vessel measurement in IAA subtypes.

Echocardiographic parameter	Type A (*n* = 16)	Type B (*n* = 15)	Type C (*n* = 5)	*p* value
Z-score of LVD	−1.58 (−2.83, −0.75)	−1.38 (−2.75, −0.84)	−2.21 (−3.62, −0.94)	0.231
Z-score of RVD	−1.39 (−2.62, 0.31)	−1.22 (−1.90, 0.38)	−2.55 (−2.87, −0.77)	0.161
LV/RV	0.80 (0.71, 1.07)	0.91 (0.77, 0.96)	0.84 (0.77, 0.94)	0.252
VSD/AO	1.47 (1.15, 2.04)	1.50 (1.40, 2.14)	1.50 (1.47, 1.67)	0.626
Z-score of AVA	−3.09 (−4.44, −1.93)	−3.64 (−4.45, −2.09)	−3.85 (−4.08, −2.42)	0.56
Z-score of PVA	2.05 (−2.41, 2.98)	1.25 (−1.10, 2.67)	1.64 (1.07, 1.81)	0.189
Z-score of MPA	1.18 (0.41, 1.92)	0.89 (0.12, 1.89)	0.96 (0.83, 2.15)	0.539
Z-score of AA	−3.51 (−4.58, −2.09)	−4.34 (−4.97, −3.05)	−2.96 (−4.19, −2.43)	0.579
Z-score of DAO	−0.29 (−3.57, 1.00)	0.24 (−0.41, 0.96)	0.35 (−1.49, 1.28)	0.618
Z-score of DA	1.30 (0.42, 1.64)	0.85 (0.15, 1.23)	−0.80 (−0.89, 2.00)	0.604
AO/PA	0.50 (0.41, 0.56)	0.44 (0.40, 0.53)	0.45 (0.45, 0.51)	0.701

### 3.3 Associated intracardiac, extracardiac, and genetic findings

Of the 72 patients with aortic arch obstruction, 64 had other abnormalities, including 36 and 28 cases of IAA and CoA, respectively. The 36 cases of IAA had other intracardiac malformations, including ventricular septal defects (*n* = 34), persistent left superior vena cava (*n* = 8), double-outlet right ventricle (*n* = 1), single ventricle with double outlet right ventricle (*n* = 1), right subclavian artery (*n* = 2), common arterial trunk (*n* = 3), main pulmonary artery septal defect (*n* = 1), and complete pulmonary vein ectopic drainage (*n* = 1). There were eight cases of combined extracardiac malformations, including hydrocephalus of the nape (n = 1), dysplasia of the corpus callosum (*n* = 1), two cases of cerebellar vermiform process defects (*n* = 2), thymus hypoplasia (*n* = 3), and an undeveloped gallbladder (*n* = 2). In the CoA group, 8 cases (22.22%) were isolated and 28 cases (77.78%) were non-isolated. Nineteen cases in the CoA group had other intracardiac malformations, including ventricular septal defects (*n* = 14), pulmonary valve stenosis (*n* = 1), complete transposition of the great arteries (*n* = 1), left heart hypoplasia (*n* = 1), bicuspid aortic valve (*n* = 5), and persistent left superior vena cava (*n* = 3). Furthermore, of the 19 cases, 15 were combined with extracardiac malformations, including single umbilical artery (*n* = 3), hypospadias (*n* = 2), nasal bone dysplasia (*n* = 2), and portosystemic shunt (intrahepatic type IV) (*n* = 1). The incidence of intracardiac and extracardiac anomalies in the IAA group was significantly higher than in the CoA group (*p* < 0.001; Table 1). The probability of interventricular septal defects was significantly higher in the IAA group than in the CoA group (*p* < 0.001) at 94.4 and 38.9%, respectively.

Genetic testing was performed in 15 of the 36 cases of IAA in this group. Of the 15 cases, 12 had no abnormalities, and 3 cases showed genetic abnormalities (20.00%, 3/15), including one case of 45XO, one case of 22q11.21 chromosomal deletion, and one case of trisomy 18 syndrome. Genetic testing was performed in 14 of the 36 cases of CoA; 11 cases had no abnormalities, whereas 3 showed genetic abnormalities (21.43%, 3/14). There was no significant difference between the IAA and the CoA group in the incidence of chromosomal abnormalities (*p* = 1.000).

### 3.4 Postpartum echocardiographic and CTA findings in aortic arch obstruction

Eight cases of IAA were examined by echocardiography after birth, of which the diagnoses in six cases were consistent with the prenatal ultrasound diagnosis and only two were misdiagnosed. In one of the misdiagnosed cases, the prenatal ultrasound diagnosis was of a double-outlet right ventricle, ventricular septal defect, and pulmonary artery crossing, which was tentatively diagnosed as the Taussig–Bing syndrome. However, the postnatal echocardiography confirmed IAA type A. In the other misdiagnosed case, the prenatal diagnosis was a narrow internal aortic arch diameter. However, the patient was diagnosed with severe CoA with collateral vascular formation on postpartum ultrasound. The final diagnosis was IAA type A following further CTA examination and surgical treatment. Among children with CoA, 36 underwent echocardiography after birth, and 31 of these diagnoses were consistent with the prenatal ultrasound diagnosis. Among the five false-negative CoA cases, three showed no abnormalities on prenatal ultrasound. Notably, one of these three cases was postnatally diagnosed with supravalvular aortic and pulmonary stenosis consistent with Williams syndrome. Of the remaining two missed CoA cases, one case showed only mild tricuspid regurgitation with a persistent left superior vena cava, and one case showed a slightly increased cardiothoracic ratio with a smaller left heart compared to the right heart, although the cause was not investigated further. Among these patients, nine underwent further CTA examinations, which confirmed the CoA diagnosis.

### 3.5 Perinatal and early clinical outcomes

Among the 36 fetuses with IAA, 28 pregnancies were terminated and 8 infants were live born. All eight live-born neonates developed early postnatal clinical deterioration, consistent with ductal-dependent systemic circulation, including cyanosis, hypoxemia, or poor systemic perfusion. Three neonates did not undergo surgical intervention and died within a few days after birth. The remaining five underwent surgical repair; among them, three had favorable early outcomes, one had an unsatisfactory postoperative course, and one died within a few days after surgery. Among the 36 live-born infants with CoA, 30 infants did not show obvious cardiovascular symptoms immediately after birth, whereas 6 developed clinical manifestations indicative of significant obstruction or ductal-dependent systemic circulation, such as dyspnea, poor perfusion, feeding difficulty, or cyanosis. Eight patients underwent surgical treatment, of which seven had good outcomes and one had poor outcome. The patient experienced complications comprising multiple organ failure, necrotizing enterocolitis (NEC), and cerebral hemorrhage post-surgery and ultimately died a few days after discharge from the hospital (Table 1). The remaining 28 infants did not undergo surgery during the available follow-up period because no definite surgical indication was documented; they were managed with clinical and echocardiographic surveillance.

We further reviewed the postnatal clinical course of the seven cases with discordant prenatal and final diagnoses, comprising five false-negative CoA cases and two prenatally misclassified IAA cases. Three cases, including one CoA and two IAA cases, received the definitive diagnosis before discharge from the maternity unit, whereas the remaining four CoA cases were diagnosed at the routine 6-week (42-day) postnatal follow-up. Among the five false-negative CoA cases, four were evaluated because of a heart murmur and one presented with dyspnea. All five had relatively mild obstruction, none required surgical intervention during the available follow-up period, and all remained in good clinical condition under clinical and echocardiographic surveillance. Both prenatally misclassified IAA cases developed dyspnea and cyanosis shortly after birth. Following postnatal cardiovascular evaluation and stabilization, both underwent successful surgical repair and remained in good clinical condition at the latest follow-up.

## 4 Discussion

In this study, fetuses with IAA and CoA were represented in equal numbers. Types A and B accounted for most cases, whereas type C was rare, a distribution consistent with previous reports (4). While both IAA and CoA were frequently associated with additional malformations, the burden and anatomical complexity of these associated defects were greater in the IAA group. Ventricular septal defect (VSD) was the most common concomitant anomaly in both groups, particularly in IAA, thereby corroborating previous reports (5–7). Rather than being dismissed as a coincidental association, this pattern may reflect a shared disturbance of conotruncal and left ventricular outflow tract development in IAA. In addition, thymic hypoplasia was primarily observed in type B IAA, a finding that is consistent with previous studies describing the close association between type B interruption and 22q11.2 deletion or DiGeorge spectrum abnormalities. Accordingly, thymic hypoplasia should not be regarded merely as an associated finding but should rather be interpreted as a clinically relevant marker prompting targeted genetic evaluation and postnatal assessment for possible immune dysfunction. In the CoA group, the frequent coexistence of a bicuspid aortic valve is likewise in line with previous studies and argues that prenatal and postnatal surveillance should extend beyond the arch lesion itself to include longitudinal monitoring of aortic valve function and progressive aortic remodeling. Taken together, these findings indicate that, once aortic arch obstruction is suspected prenatally, the examination should transition from simple structural confirmation to syndrome-oriented evaluation, including the systematic assessment of intracardiac anatomy, extracardiac anomalies, thymic morphology, and genetic risk.

IAA and CoA are the two principal forms of aortic arch obstruction, and accurate prenatal differentiation between them carries direct clinical implications for fetal circulatory dependence, postnatal urgency, and counseling priorities for families. Previous studies have primarily emphasized qualitative sonographic markers, particularly in fetal CoA, such as ventricular disproportion, isthmic narrowing, and arch hypoplasia; however, these signs alone may not provide sufficient discriminatory specificity. In this context, our results suggest that the diagnostic value of prenatal ultrasonography lies not only in recognizing arch discontinuity or localized stenosis but also in evaluating quantitative cardiovascular remodeling alongside anatomical assessment. In particular, the higher VSD-to-aorta ratio in the IAA group corresponds with previous anatomical and surgical observations that VSD is one of the most frequent associated lesions in IAA (29, 30). This finding may reflect a more profound perturbation of left ventricular outflow tract development, with redistribution of blood flow through the VSD and greater reliance on alternative fetal pathways to maintain systemic output. Similarly, the lower aortic valve annulus Z-score, ascending aortic Z-score, and aortic-to-pulmonary artery diameter ratio in IAA should be interpreted not simply as smaller measurements but as morphometric correlates of more advanced underdevelopment of the left-sided outflow pathway. This interpretation is also consistent with the fact that CoA usually retains some residual antegrade arch flow, whereas IAA represents the complete loss of arch continuity. Similarly, the higher ductus arteriosus Z-score in IAA reinforces the concept that the ductus arteriosus functions as the major compensatory route for systemic perfusion in the setting of complete interruption. The elevated LV-to-RV ratio in IAA may also indicate altered ventricular interaction secondary to severe outflow obstruction and larger septal communication rather than simple chamber size differences alone. Taken together, these observations suggest that a multiparametric approach may sharpen prenatal discrimination between IAA and CoA and may also help identify fetuses at higher risk of early postnatal ductal dependence. As intracardiac parameters did not differ significantly among IAA subtypes, subtype classification should still rely primarily on branch pattern recognition and characteristic arch morphology rather than defaulting to routine biometric indices alone.

We further investigated the characteristics of genetic abnormalities and their potential clinical associations. Previous studies indicate that IAA—particularly type B—is highly associated with the 22q11.2 microdeletion syndrome (8, 9). In our cohort, this specific deletion was observed less frequently, which may reflect either differences in the sensitivity of the genetic testing methods applied or the limited sample size associated with this rare anomaly. However, this discrepancy should be interpreted cautiously rather than as contradictory evidence because it may also reflect the limited number of genetically tested cases and the retrospective design. Nonetheless, our analysis revealed comparable overall rates of genetic abnormalities between the IAA and CoA groups. Rather than undermining the existing literature, this finding suggests that genetic evaluation remains important in both conditions, particularly when thymic hypoplasia, extracardiac anomalies, or complex intracardiac defects are present. Accordingly, both diagnoses warrant careful prenatal genetic counseling and consideration of chromosomal microarray analysis in clinically selected cases. Notably, the identification of a rare case of IAA associated with Turner syndrome extends the current understanding of the genetic etiology of arch obstructions. While Turner syndrome is classically associated with left-sided obstructive lesions such as CoA, its occurrence as an interrupted arch may broaden the phenotypic boundaries of aortic arch obstruction and raise the possibility that sex chromosome abnormalities can also intersect with more severe forms of arch maldevelopment.

Echocardiography is a routine postnatal diagnostic method for aortic arch obstruction. However, it has some limitations. The diagnostic capability of echocardiography may be insufficient in cases of aortic arch obstruction with complex anatomical variations. Furthermore, echocardiography may not detect smaller or earlier aortic stenoses. However, CTA can provide more comprehensive information, particularly regarding the display of the aortic arch, descending aorta, and surrounding structures. CTA can show the anatomical morphology of the aorta, including the location and degree of aortic coarctation and the possible interruption of the aortic arch (31, 32). This function is particularly important when dealing with complex aortic arch variations, branch abnormalities, and interruptions, combined with other heart lesions. One particular case in this study is noteworthy, wherein prenatal ultrasonography revealed a small internal diameter of the aortic arch, whereas postnatal echocardiography revealed severe aortic coarctation with collateral vascular formation and a possible single coronary artery malformation. Further CTA examination revealed an IAA type A, which was consistent with the intraoperative exploration and CTA. Therefore, ultrasonography has certain limitations in the diagnosis of aortic coarctation and interruption. In some complex cases, combining other imaging methods may be important for further confirmation. Another important finding of this study is the pattern of missed and misdiagnosed prenatal cases. Our case review revealed that missed diagnoses predominantly occurred in CoA, particularly when the lesion was mild or prenatal findings were limited to subtle, indirect signs, such as slight ventricular disproportion, mild cardiomegaly, or isolated findings without clear evidence of arch narrowing. Misdiagnosis primarily occurred in complex IAA cases in which attention was drawn to major intracardiac malformations, or in cases where severe coarctation and type A IAA were difficult to distinguish based on prenatal ultrasound alone. These observations suggest that high-risk factors may increase the risk of the prenatal ultrasonic misdiagnosis or missed diagnosis of aortic arch obstruction: (1) mild or evolving obstruction without typical hemodynamic changes during fetal life, (2) reliance on indirect signs without a systematic assessment of aortic arch continuity and isthmus morphology, (3) diagnostic distraction caused by associated cardiac or extracardiac anomalies, and (4) difficulty differentiating severe CoA from IAA in anatomically complex cases. Therefore, when subtle left–right asymmetry, unexplained ventricular disproportion, a persistent left superior vena cava, or other associated anomalies are present, a targeted reassessment of the three-vessel-trachea view, the aortic arch long-axis view, and ductal arch continuity should be performed. Serial follow-up or multimodality postnatal imaging should also be considered when necessary. Our review of the prenatally discordant cases illustrates the heterogeneous clinical consequences of an incorrect or missed prenatal diagnosis. The five false-negative CoA cases had relatively mild obstruction and remained clinically stable without surgical intervention during follow-up. In contrast, the two prenatally misclassified IAA cases developed early respiratory distress and cyanosis and required prompt postnatal stabilization and surgical repair. These observations suggest that accurate prenatal recognition of severe ductal-dependent lesions may facilitate lesion-specific delivery planning and early neonatal management. However, because of the small number of discordant cases, differences in lesion severity, and the retrospective design, the effect of prenatal diagnostic status on morbidity and survival could not be determined.

IAA and CoA differ fundamentally in postnatal pathophysiology. IAA represents complete anatomical discontinuity of the aortic arch and is therefore a profoundly ductal-dependent lesion. Without maintenance of ductal patency and timely surgical intervention, systemic perfusion may deteriorate rapidly after ductal constriction or closure, and survival is generally not expected. Therefore, prenatal recognition of IAA should prompt planned delivery at a tertiary pediatric cardiac center, immediate neonatal cardiovascular assessment, prostaglandin infusion when indicated, and early surgical repair. In our cohort, neonates with IAA universally developed hypoxia and cyanosis shortly after birth, and mortality was high in those who did not undergo surgery. This observation is consistent with previous neonatal and surgical series (33). Even when surgery was performed, outcomes remained less favorable than those observed in CoA. In contrast, CoA exhibits a broader spectrum of postnatal severity because anatomical continuity between the ascending and descending aorta is preserved. Isolated CoA does not typically present primarily with hypoxia. Symptomatic neonates with severe CoA may instead develop poor systemic perfusion, heart failure, metabolic acidosis, shock, or ductal-dependent systemic circulation, depending on the degree of obstruction and ductal status. This clinical heterogeneity explains why some CoA cases require urgent intervention, whereas others may be managed initially with close clinical and echocardiographic surveillance (34). This relative hemodynamic reserve may explain why CoA often has a broader diagnostic and therapeutic window, but it also highlights a recurrent diagnostic problem: Clinically milder presentation may contribute to delayed recognition. In this context, prenatal diagnosis remains valuable because it may improve preoperative status and reduce early morbidity. Overall, these divergent mechanisms support lesion-specific perinatal management strategies: IAA requires an integrated prenatal-postnatal management pathway, ideally including delivery at a tertiary cardiac center and rapid neonatal stabilization, whereas CoA may be managed with risk stratification, close surveillance, and timely intervention according to symptom progression and imaging confirmation.

## 5 Limitations

The limitations of this study include a small sample size, which may affect statistical power and the detection of rare variants. These limitations affect the overall understanding of the relationship between aortic arch obstruction and genetic abnormalities. Future studies should increase the sample size and utilize gene editing technology and big data analysis to further explore the genetic mechanisms that underlie aortic arch obstruction. Another limitation is the clinical heterogeneity within the CoA cohort. The included CoA cases comprised both isolated and non-isolated lesions and showed variable postnatal severity. Due to the retrospective design and limited sample size, a more detailed subgroup analyzes according to anatomical severity or associated anomalies were not performed. In addition, only five false-negative CoA cases and two prenatally misclassified IAA cases were identified. The small sample size, differences in lesion severity, and retrospective availability of clinical data precluded an adequately powered comparison of morbidity and prognosis between prenatally diagnosed and discordant cases.

## 6 Conclusion

Prenatal echocardiography is valuable for the detection, differentiation, and perinatal risk stratification of fetal aortic arch obstruction. Compared with CoA, IAA was associated with more severe left-sided outflow tract underdevelopment, a higher burden of intracardiac anomalies, greater ductal dependence, and poorer early outcomes. A structured prenatal assessment integrating arch morphology, quantitative cardiovascular parameters, associated anomalies, genetic evaluation, and planned postnatal management may improve counseling and facilitate timely lesion-specific intervention.

## Data Availability

The original contributions presented in the study are included in the article/supplementary material, and further inquiries can be directed to the corresponding author/s.
